# Meta-analyses between 18 candidate genetic markers and overweight/obesity

**DOI:** 10.1186/1746-1596-9-56

**Published:** 2014-03-12

**Authors:** Linlin Tang, Huadan Ye, Qingxiao Hong, Fei Chen, Qinwen Wang, Leiting Xu, Shizhong Bu, Qiong Liu, Meng Ye, Dao Wen Wang, Yifeng Mai, Shiwei Duan

**Affiliations:** 1 Zhejiang Provincial Key Laboratory of Pathophysiology, School of Medicine, Ningbo University, 315211 Ningbo, Zhejiang, China; 2 The Affiliated Hospital, Ningbo University, 315000 Ningbo, Zhejiang, China; 3 Diabetes Center, School of Medicine, Ningbo University, Ningbo 315211, Zhejiang, China; 4 Institute of Hypertension and Department of Internal Medicine, Tongji Hospital, Tongji Medical College, Huazhong University of Science and Technology, Wuhan, China

**Keywords:** *SH2B1*, *FAIM2*, Polymorphism, Overweight, Obesity, Meta-analysis

## Abstract

**Aims:**

The goal of our study is to investigate the associations between 18 candidate genetic markers and overweight/obesity.

**Methods:**

A total of 72 eligible articles were retrieved from literature databases including PubMed, Embase, SpingerLink, Web of Science, Chinese National Knowledge Infrastructure (CNKI), and Wanfang. Meta-analyses of 18 genetic markers among 56,738 controls and 48,148 overweight/obese persons were done by Review Manager 5.0.

**Results:**

Our results showed that *SH2B1* rs7498665 polymorphism was significantly associated with the risk of overweight/obesity (overall odds ratio (OR) = 1.21, 95% confidence interval (CI) = 1.09-1.34, P = 0.0004). Increased risk of overweight/obesity was also observed in *FAIM2* rs7138803 polymorphism (overall OR = 1.11, 95% CI = 1.01-1.22, P = 0.04).

**Conclusion:**

Our meta-analyses have shown the important role of 2 polymorphisms (*SH2B1* rs7498665 and *FAIM2* rs7138803) in the development of overweight/obesity. This study highlighted the importance of above two candidate genes (*SH2B1* and *FAIM2*) in the risk of overweight/obesity.

**Virtual slides:**

The virtual slide(s) for this article can be found here: http://www.diagnosticpathology.diagnomx.eu/vs/2785487401176182.

## Introduction

Overweight/obesity as a metabolic disorder is closely associated with diabetes mellitus and cardiovascular disease, which are chronic diseases influencing the average life expectancy [1,2]. In 2008, world health organization (WHO) has reported that a large portion of adults (>20 yr) were overweight (35%) and obese (12%) [3]. The overweight/obesity will become an epidemic [4] and cause a huge economic burden of society [4] in the near future.

The occurrence and the development of obesity are influenced by both environmental and genetic factors [5,6]. Environmental factors, such as poor nutritional state and a lack of physical exercise, have an impact on the development of overweight/obesity [7,8] through the epigenetic modifications such as gene methylation [9]. Genetic polymorphisms can confer the susceptibility of overweight/obesity and obesity-related morbidities [10]. Recent genome-wide association studies (GWAS) have identified a handful of candidate genetic markers to the risk of overweight/obesity [11].

In the present study, we performed a systematic search for eligible studies in the meta-analyses. Our results identified 18 polymorphisms among 16 genes that were all the candidate genes of obesity. Among these genes, *GNB3* encodes β3-subunit protein which is involved in the process of hypertension and obesity [12]. *MTHFR* gene encodes methylenetetrahydrofolate reductase that is shown to be associated with increased fasting homocysteine [13]. *MTHFR* polymorphism is shown to be associated with lipid metabolism in the elderly women [14]. *CNR1* is shown to regulate the endocannabinoid system that might stimulate the metabolism of lipogenesis through central and peripheral mechanisms [15,16]. *CNR1* is associated with low HDL dyslipidemia and a common haplotype of *CNR1* could be a protective factor of obesity-related dyslipidemia [17]. *BDNF* is shown to play an important role in the development of several neuronal systems [18]. As an effector on energy homeostasis through MC4R signaling pathway, *BDNF* has an effect on the glucose and lipid metabolism in obese diabetic animals [19,20]. *FAAH* gene encodes fatty acid amide hydrolase [21] and plays an important role in the development of obesity [22]. *ADRB1* is shown to mediate in lipolysis and thus is important for obesity [23]. Rat study identifies that *ADRB1* mediates the sympathetic nervous system (SNS) stimulation of thermogenesis in brown adipose tissue [24]. *SH2B1* is able to bind leptin to its receptor, and thus increases the JAK2 activation which is involved in the insulin and leptin signaling [25,26]. *PCSK1* encodes prohormone convertase 1/3 that is a vital enzyme in the regulation of a majority of neuroendocrine body weight control [27]. A novel homozygous missense mutation in *PCSK1* leads to early-onset obesity [28]. *NPY2R* is a presynaptic receptor [29] playing an inhibitory role in the control of appetite regulation [30], and thus influences the development of obesity [31]. *FAIM2* (Fas apoptotic inhibitory molecule 2) is an anti-apoptotic gene [32]. Mutations of *FAIM2* which interferes with Fas-mediated cell death confer risk for obesity [33]. *SERPINE1* encodes a member of serine proteinase inhibitor which influences plasma PAI-1 activity with relation to obesity [34]. Serum paraoxonase-1 (PON1) encoded by *PON1* as an enzyme associated with HDL-C could be a protector against oxidative damage in obesity [35]. *CETP* protein product transfers cholesterylesters from HDL to pro-atherogenic apoB-lipoproteins and thus has an impact on the lipid and HDL metabolism [36,37]. *UCP1* encodes uncoupling protein 1 that is mediated by long-chain fatty acids (LCFAs) from brown adipose tissue [38]. *UCP1* expression in adipose tissue has an impact on regulating the thermogenesis and lipolysis [39,40]. Mitochondrial uncoupling by UCP1 has demonstrated to be a target in antiobesity therapies [41]. *ABCA1* gene product mediates the transport of cholesterol, phospholipids, and other metabolites [42]. Exercise has an impact on *ABCA1* expression along with increased HDL levels in obese boys [43]. *APOE* plays a fundamental role with ligand-receptor in uptaking lipoproteins, and thus participates in the lipid metabolism [44]. In addition, *APOE* correlates with inflammation in adipose tissue in high-fat diet-induced obesity [45].

Meta-analysis is a systematic evaluation by combining the results from collected studies [46,47]. The major advantages of meta-analysis are to improve the precision and accuracy by pooling up the data from multiple sources, and to analyze and quantify the inconsistency of results and the publish bias [48]. In the present study, we conducted comprehensive meta-analyses to identify the contribution of 18 polymorphisms to overweight/obesity.

## Materials and methods

### Literature search and data extraction

We performed the literature research using related databases such as PubMed, Embase, SpingerLink, Web of Science, Chinese National Knowledge Infrastructure (CNKI), and Wanfang. The combination of keywords in the literature search was obesity or overweight together with polymorphism or mutation or variant or single nucleotide polymorphism (SNP). The studies excluded in the meta-analysis met the following criteria: (1) the study had been included in the previous meta-analysis; (2) the study was not involved with genetic testing; (3) the study was not a case–control study. The criteria for overweight or obesity in adolescents and children were defined by WHO [49,50]. Finally, we harvested 18 polymorphisms of 16 genes in the current meta-analysis. These included *GNB3* rs5443, *MTHFR* rs1801133, *CNR1* rs806381, *BDNF* rs6265, *FAAH* rs324420, *ADRB1* rs1801253, *SH2B1* rs7498665, *PCSK1* rs6232 and rs6235, *NPY2R* rs1047214, *FAIM2* rs7138803, *SERPINE1*rs1799768, *PON1* rs854560 and rs662, *CETP* TaqIB, *UCP1* rs1800592, *ABCA1* rs2230806 and *APOE* ϵ2/ϵ3/ϵ4.

#### Statistical analysis

Meta-analysis was performed by using Statistical software Review Manager 5.0 [51]. Forest plots included the ORs with the corresponding 95% CIs, cochran’s Q and the inconsistency index (I^2^). If there were no significant heterogeneity (I^2^ < 50%, P > 0.05) of the studies in the meta-analysis, we used a fixed-effect model for the analysis. Otherwise, a random-effect model was used for the meta-analysis with large heterogeneity (I^2^ > 50%, P < 0.05). The weight of each involved study was calculated whatever in fixed-effect or random-effect model in forest plots by Review Manager 5.0. Two tailed P value < 0.05 was treated as significant. Power analyses were calculated by Power and Sample Size Calculation software (v3.0.43) [52].

## Results

An initial search returned a total of 7,750 literatures from databases including PubMed, Embase, SpingerLink, Web of Science, Chinese National Knowledge Infrastructure (CNKI), and Wanfang. After a systematic filtration, 72 eligible articles, including 64 English, 6 Chinese, 1 German and 1 Spanish articles, were left for the meta-analyses (Additional file 1: Table S1). The detailed information for the retrieved studies was shown in Tables 1 and 2.

**Table 1:** Characteristics of 17 single nucleotide polymorphisms

**Gene**	**SNP**	**Year**	**Author**	**Race**	**Cases/Controls (n)**	**Allele 1**	**Allele 2**	**Model selected**	**Heterogeneity**	**P value**	**Odds ratio (95% confidence interval)**
						**(Case/Controls, n)**	**(Case/Controls, n)**		**(I2)%**		
*GNB3*	rs5443	1999	Siffert W	Caucasian	92/207	108/392	76/122	Fixed	42	0.47	1.04 (0.93-1.16)
	(C/T)	1999	Siffert W	Asian Chinese	186/832	166/886	206/778				
		1999	Siffert W	African	127/607	34/219	220/995				
		2000	Siffert W	Caucasian	207/92	292/108	122/76				
		2001	Hinney A	Caucasian	491/330	695/442	287/218				
		2001	Benjafield AV	Caucasian	92/188	133/284	51/92				
		2001	Ohshiro Y	Asian Japanese	208/150	215/148	201/152				
		2004	Suwazono Y	Asian Japanese	505/2120	517/2177	493/2063				
		2008	Wang X	Asian Chinese	129/270	442/285	376/255				
		2013	Hsiao TJ	Asian Chinese	467/505	402/441	532/569				
*MTHFR*	rs1801133	2007	Terruzzi I	Caucasian	84/52	90/61	78/43	Fixed	0	0.59	1.05 (0.87-1.27)
	(C/T)	2010	Tavakkoly Bazzaz J	Asian Iranian	74/207	109/306	39/108				
		2012	Yin RX	Asian Chinese	751/978	1049/1383	453/573				
*CNR1*	rs806381	2008	Benzinou M	Caucasian	839/1726	1163/2362	515/1090	Fixed	0	0.5	1.04 (0.93-1.17)
	(A/G)	2008	Jaeger JP	Caucasian	430/317	613/464	247/170				
		2012	Zhuang M	Asian Chinese	1662/1070	2345/1550	979/590				
*BDNF*	rs6265	2005	Friedel S	Caucasian	183/283	342/448	81/118	Fixed	46	0.8	1.01 (0.92-1.11)
	(G/A)	2009	Hotta K	Asian Japanese	1127/1733	1367/2013	887/1453				
		2009	Marti A	Caucasian	155/147	242/226	68/68				
		2011	Xi B	Asian Chinese	1229/1619	1095/1554	1363/1684				
		2011	Rouskas K	Caucasian	510/469	826/732	194/206				
		2012	Skledar M	Caucasian	74/226	111/374	37/78				
*FAAH*	rs324420	2005	Sipe JC	Caucasian	1094/1594	1777/984	411/204	Random	79	0.54	0.94 (0.76-1.16)
	(C/A)	2005	Sipe JC	African	507/107	687/161	327/53				
		2005	Sipe JC	Asian	271/94	471/148	71/40				
		2007	Jensen DP	Caucasian	4190/2507	6817/3991	1563/1023				
		2008	Durand E	Caucasian	1517/1320	2473/2104	561/536				
		2008	Papazoglou D	Caucasian	158/121	265/209	51/33				
		2008	Moneletone P	Caucasian	378/110	614/194	142/26				
		2010	Muller TD	Caucasian	2818/2818	3027/4607	689/1029				
*ADRB1*	rs1801253	2001	Rydén M	Caucasian	141/157	206/214	76/100	Fixed	0	0.5	1.03 (0.94-1.14)
	(C/G)	2004	Tafel J	Caucasian	296/134	403/180	189/88				
		2007	Gjesing AP	Caucasian	4575/3073	6781/4609	2369/1537				
		2008	Ohshiro Y	Asian Japanese	180/132	284/215	76/49				
*SH2B1*	rs7498665	2009	Hotta K	Asian Japanese	1129/1735	1943/3003	315/467	Fixed	0	0.0004	1.21 (1.09-1.34)
	(A/G)	2010	Shi J	Asian Chinese	829/1859	1427/3317	231/401				
		2011	Beckers S	Caucasian	1045/317	1223/401	867/223				
		2011	Rouskas K	Caucasian	510/469	673/675	347/263				
		2012	Volckmar AL	Caucasian	3139/424	3728/557	2550/311				
*PCSK1*	rs6232	2009	Happé F	Caucasian	3570/7933	6735/15028	405/838	Fixed	34	0.08	1.14 (0.97-1.12)
	(A/G)	2011	Rouskas K	Caucasian	510/469	969/882	51/56				
		2012	Villalobos-Comparán M	South American Mexican	1018/1364	2005/2709	31/19				
		2013	Choquet H	European American	263/547	485/1041	41/53				
		2013	Dušátková L	Asian Czech	668/770	1255/1469	81/71				
*PCSK1*	rs6235	2009	Happé F	Caucasian	3559/7793	5164/11432	1954/4154	Fixed	0	0.26	1.04 (0.97-1.12)
	(G/C)	2012	Villalobos-Comparán M	South America Mexican	994/1336	1575/2156	413/516				
		2013	Choquet H	European - American	263/547	368/793	158/301				
		2013	Choquet H	African - American	453/251	740/432	166/70				
		2013	Dušátková L	Asian Czech	670/772	996/1130	344/414				
		2014	Hsiao TJ	Asian Chinese	290/175	406/229	174/121				
*NPY2R*	rs1047214	2006	Torekov SS	Caucasian	939/4767	1026/5295	852/4239	Fixed	0	0.54	0.97 (0.88-1.07)
	(T/C)	2007	Siddiq A	Caucasian	953/1042	1048/1132	858/952				
		2007	Wang HJ	Caucasian	184/183	189/169	179/197				
		2009	Zhang J	Asian Chinese	705/1325	1171/2133	239/517				
*FAIM2*	rs7138803	2009	Hotta K	Asian Japanese	1125/1726	1408/2251	842/1201	Fixed	0	0.04	1.11 (1.01-1.22)
	(G/A)	2011	Xi B	Asian Chinese	1229/1619	1711/2332	747/906				
		2011	Rouskas K	Caucasian	510/469	643/610	377/328				
		2013	Li C	Asian Chinese	242/469	331/663	153/275				
		2013	Zhao XY	Asian Chinese	371/393	534/565	208/221				
*SERPINE1*	rs1799768	2001	Sartori MT	Caucasian	93/79	95/84	91/74	Fixed	39	0.07	0.83 (0.67-1.02)
	(4G/5G)	2002	Hoffstedt J	Caucasian	317/188	305/141	329/235				
		2006	Berberoğlu M	Asian Turk	126/133	151/133	101/133				
		2008	Solá E	Caucasian	67/67	70/65	64/69				
		2008	Kinik ST	Asian Turk	39/38	52/36	26/40				
		2011	Espino A	South American Chilean	50/71	32/51	44/52				
		2012	Wingeyer SD	South American Argentine	110/111	92/109	128/113				
*PON1*	rs854560	2011	Veiga L	Caucasian	81/74	101/90	61/58	Fixed	31	0.4	0.87 (0.62-1.21)
	(A/T)	2011	Martínez-Salazar MF	South American Mexican	63/64	114/101	12/27				
		2013	Rupérez AI	Caucasian	177/81	210/219	137/143				
*PON1*	rs662	2011	Veiga L	Caucasian	81/74	68/44	94/104	Fixed	18	0.6	1.09 (0.79-1.51)
	(G/A)	2011	Martínez-Salazar MF	South American Mexican	63/64	66/65	60/63				
		2013	Rupérez AI	Caucasian	177/81	252/249	102/111				
*CETP*	TaqIB	2006	Huang ZY	Asian Chinese	199/141	243/162	155/120	Fixed	0	0.23	0.91 (0.79-1.06)
	(B1/B2)	2008	Srivastava N	Asian Indian	159/278	153/263	165/293				
		2010	Ruan X	Asian Chinese	934/924	1104/1028	764/820				
		2011	Huang Y	Asian Chinese	206/132	250/155	162/109				
*UCP1*	rs1800592	1998	Gagnon J	Caucasian	674/311	1013/473	335/149	Random	60	0.23	1.19 (0.90-1.57)
	(A/G)	2000	Proenza AM	Asian Turk	136/94	189/131	83/57				
		2002	Kieć-Wilk B	Caucasian	12/106	18/146	6/66				
		2002	Nieters A	Caucasian	154/153	232/231	76/75				
		2003	Forga Ll	Caucasian	159/154	258/244	60/64				
		2004	Ramis JM	Caucasian	82/170	259/433	49/81				
		2008	Mottagui-Tabar S	Caucasian	91/479	433/736	149/222				
		2009	Shen ZN	Asian Chinese	127/257	129/240	125/274				
*ABCA1*	rs2230806	2006	Porchay I	Caucasian	2097/2947	2992/4238	1202/1656	Fixed	0	0.87	1.01 (0.90-1.13)
	(G/A)	2007	Kitjaroentham A	Asian Thai	112/117	143/143	81/91				
		2011	Huang Y	Asian Chinese	206/132	233/141	179/123				

**Table 2:** **Characteristics of *APOE *** **ϵ2/ϵ3/ϵ4 polymorphism**

**Year**	**Author**	**Race**	**Case/Controls (n)**	**Genotypes (case/controls, n)**						**Alleles (case/controls, n)**		
				**ϵ2/ϵ2**	**ϵ2/ϵ3**	**ϵ2/ϵ4**	**ϵ3/ϵ3**	**ϵ3/ϵ4**	**ϵ4/ϵ4**	**ϵ2**	**ϵ3**	**ϵ4**
2003	Guerra A	Caucasian	31/81	0/0	6/4	0/0	63/20	13/7	0/0	6/4	145/51	13/7
2008	Srivastava N	Asian Indian	159/278	0/1	17/18	2/6	90/198	41/55	9/0	19/30	238/469	61/61
2010	Ergun MA	Asian Chinese	38/42	0/2	2/0	12/4	8/9	16/26	0/1	14/8	34/44	28/32
2012	Zhang J	Asian Chinese	282/172	1/3	46/16	7/2	186/123	40/27	2/1	55/24	458/289	51/31
2012	Zarkesh M	Asian Iran	463/370	1/1	48/38	6/7	348/268	63/53	3/3	56/47	807/627	75/66
**Module**	**Case/Controls (n)**	**Model selected**	**Heterogeneity (I2)%**	**P value**	**OR (95% CI)**							
ϵ2/ϵ2/ϵ3/ϵ3	954/813	Fixed	0	0.12	0.35 (0.09-1.32)							
ϵ2/ϵ3ϵ3/ϵ3	814/694	Fixed	48	0.07	1.33 (0.98-1.82)							
ϵ2/ϵ4/ϵ3/ϵ3	695/618	Fixed	0	0.92	0.96 (0.45-2.05)							
ϵ3/ϵ4/ϵ3/ϵ3	868/786	Fixed	28	0.7	1.05 (0.82-1.35)							
ϵ4/ϵ4/ϵ3/ϵ3	695/618	Random	63	0.54	1.89 (0.25-14.46)							
ϵ2/ϵ3	1832/1593	Fixed	23	0.26	1.16 (0.90-1.51)							
ϵ4/ϵ3	1910/1681	Random	65	0.54	1.13 (0.77-1.66							

**Figure 1:**
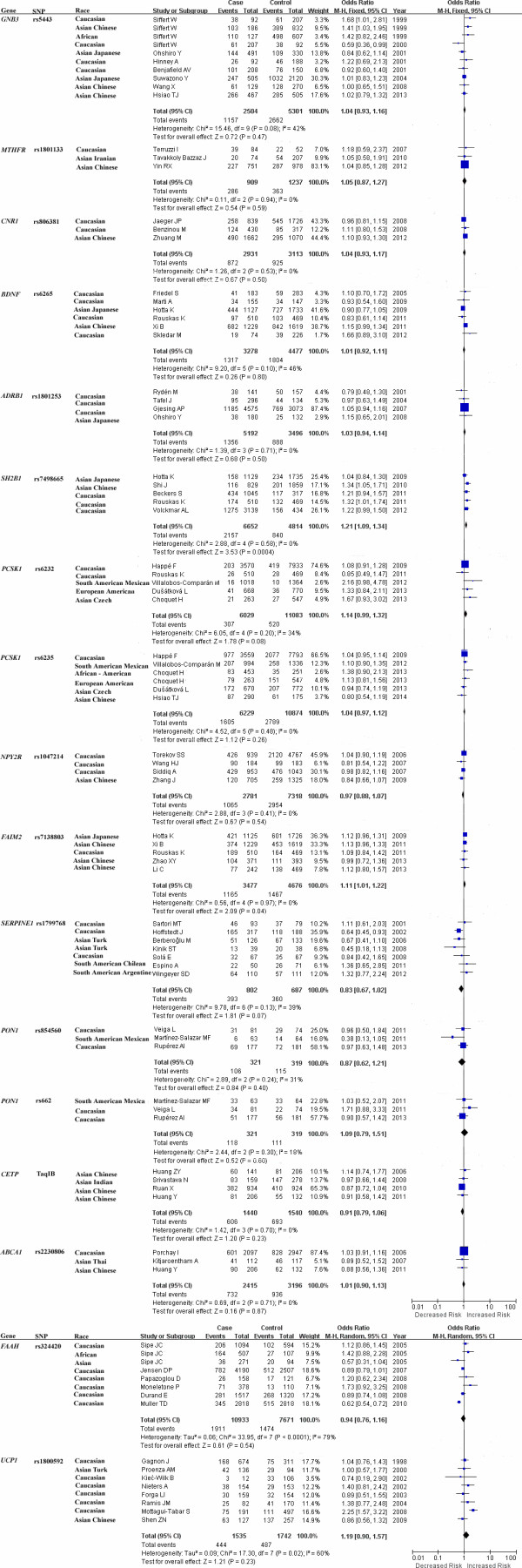
Forest plots of the association studies between 17 SNPs and overweight/obesity.

**Figure 2:**
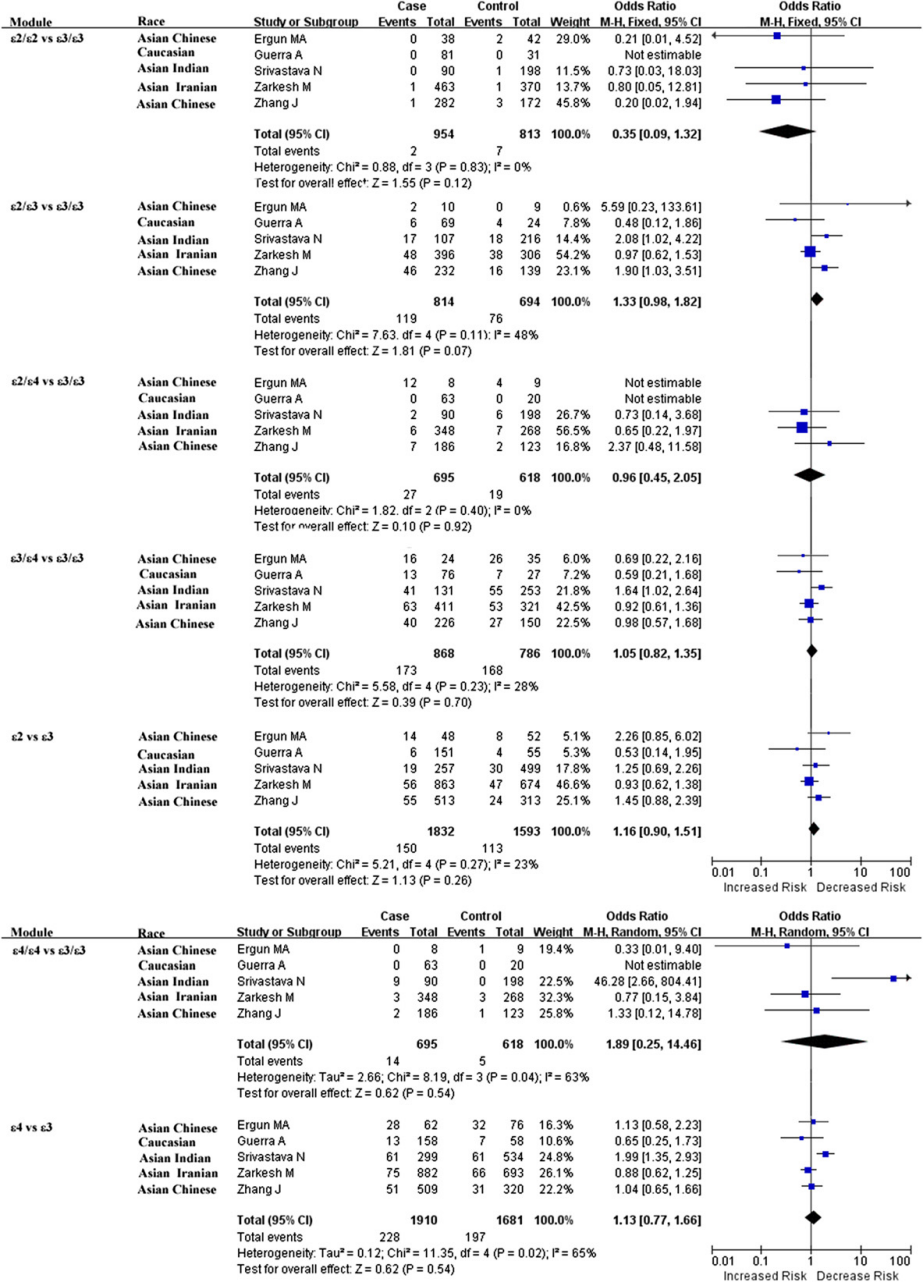
**Forest plots of the association studies between *APOE *** **ϵ2/ϵ3/ϵ4 polymorphism and overweight/obesity.**

**Figure 3:**
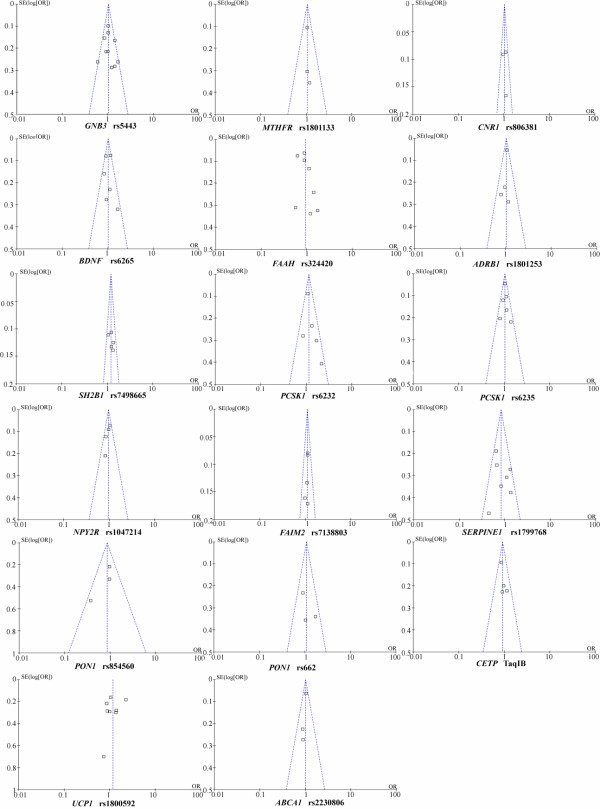
Funnel plots of the studies of 17 SNPs involved in meta-analysis.

**Figure 4:**
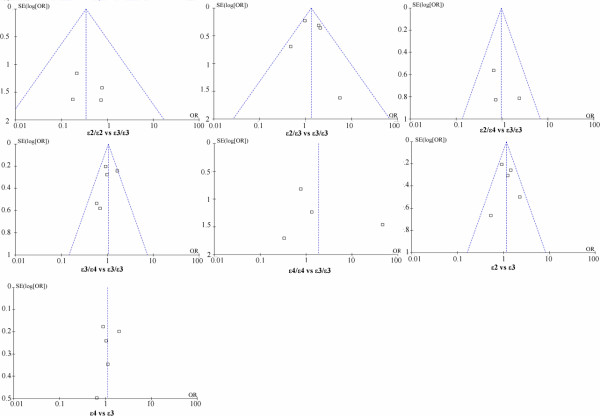
**Funnel plots of the studies of *APOE *** **ϵ2/ϵ3/ϵ4 involved in meta-analysis.**

Heterogeneity is an important indicator to identify if there is difference in the collected studies. According to the extent of heterogeneity, we categorized the meta-analyses into three groups that have minimal (I^2^ = 0), moderate (I^2^ < 50%), and significant heterogeneity (I^2^ ≥ 50%), respectively. As shown in Figure 1, minimal heterogeneity (I^2^ = 0) was found for the meta-analyses of 10 polymorphisms that included *MTHFR* rs1801133, *CNR1* rs806381, *ADRB1* rs1801253, *SH2B1* rs7498665, *PCSK1* rs6235, *NPY2R* rs1047214, *FAIM2* rs7138803, *CETP* TaqIB and *ABCA1* rs2230806. Moderate heterogeneity was found for 5 polymorphisms, including *BDNF* rs6265 (I^2^ = 46%), *PCSK1* rs6232 (I^2^ = 34%), *GNB3* rs5443 (I^2^ = 42%), *PON1* rs854560 (I^2^ = 31%), *PON1* rs662 (I^2^ = 18%), and *SERPINE1* rs1799768 (I^2^ = 39%). Significant heterogeneity was found for *UCP1* rs1800592 (I^2^ = 60%) and *FAAH* rs324420 (I^2^ = 79%). Moreover, As shown in Figure 2, various heterogeneities were shown in the meta-analyses of *APOE* ϵ2/ϵ3/ϵ4 polymorphism under the seven genetic models (ϵ2/ϵ3 versus ϵ3/ϵ3: I^2^ = 48%; ϵ2/ϵ4 versus ϵ3/ϵ3: I^2^ = 0%; ϵ3/ϵ4 versus ϵ3/ϵ3: I^2^ = 28%; ϵ4/ϵ4 versus ϵ3/ϵ3: I^2^ = 63%; ϵ2/ϵ3 versus ϵ3/ϵ3: I^2^ = 0%; ϵ2 versus ϵ3: I^2^ = 23%; ϵ4 versus ϵ3: I^2^ = 65%). No obvious publication bias was observed based on their funnel plots (Figures 3 and 4).

Our results showed that *SH2B1* rs7498665 was significantly associated with the risk of overweight/obesity among 6,142 cases and 4,345 controls from four studies (overall OR = 1.21, 95% CI = 1.09-1.34, P = 0.0004, Figure 1). Increased risk of overweight/obesity was also observed in rs7138803 of *FAIM2* among 3,477 cases and 4,676 controls from five studies (overall OR = 1.11, 95% CI = 1.01-1.22, P = 0.04, Figure 1). No evidence of association was observed for the meta-analyses of the rest 16 variants (Figures 1 and 3). For the meta-analyses with large heterogeneity, we further performed subgroup meta-analyses by ethnicity. No significant association of *UCP1* rs1800592 with overweight/obesity was observed in Caucasian (P = 0.13, I^2^ = 62%), and Asian (P = 0.59, I^2^ = 0%, Additional file 2: Figure S1). And the subgroup meta-analysis of *APOE* ϵ2/ϵ3/ϵ4 polymorphism by excluding the study of Srivastava et al. [53] didn’t produce any significant association of *APOE* ϵ2/ϵ3/ϵ4 with overweight/obesity (Additional file 3: Figure S2). There was no visual publication bias in all the above meta-analyses (Additional file 4: Figure S3).

## Discussion

Current meta-analyses were performed among 48,148 cases and 56,738 controls from 72 studies, covering a total of 6 populations, including Caucasian, Asian, Japanese-American, European-American, African-American, South American, and African. Among the tested 18 polymorphisms, there were two (*SH2B1* rs7498665 and *FAIM2* rs7138803) with significant association results (P < 0.05). Power analysis also showed large power existed in our meta-analyses of two significant polymorphisms including *SH2B1* rs7498665 (100%) and *FAIM2* rs7138803 (100%).

*SH2B1* encodes an adaptor protein associated with leptin and insulin signaling in the lipid metabolism [54]. *SH2B1* is an enhancer that may influence the phenotype of obesity through JAK-STAT pathway [55], which is important in the development and function of adipocytes [56]. *SH2B1* acts as a mediator through PI3-kinase pathway which is correlated with the biological actions of leptin [26]. Many animal studies have shown that *SH2B1* is involved in the development of obesity. *SH2B1* through its participation in the regulation of leptin sensitivity, energy metabolism and body weight [57]. *SH2B1* has been identified to be related to obesity through genome-wide association studies (GWAS) [55]. Our meta-analysis of *SH2B1* rs7498665 was performed among 6,652 cases and 4,814 controls with four studies. Among the tested populations, no heterogeneity was observed (I^2^ = 0). Our results confirmed the relationship between *SH2B1* and the risk of overweight/obesity (overall OR = 1.21, 95% CI = 1.09-1.34, P = 0.0004, Figure 1).

*FAIM2* is an anti-apoptotic gene that provides protection from Fas-mediated cell death [32] that is associated with extreme overweight by GWAS [58]. *FAIM2* rs7138803 polymorphism is associated with increased risk of obesity in Japanese [59]. But there is no relationship between *FAIM2* rs7138803 and obesity in Chinese [60]. Minor allele frequency of rs7138803 in Chinese populations ranges from 0.28 to 0.29, while *FAIM2* rs7138803 is monomorphic in Japanese and Caucasian populations. Our meta-analysis among 3477 cases and 4676 controls demonstrated that *FAIM2* rs7138803 was associated with the risk of overweight/obesity (overall OR = 1.11, 95% CI = 1.01-1.22, P = 0.04, Figure 1).

Although meta-analysis is an important method to improve the precision and accuracy, to analyze and quantify the published results [61-63], some disadvantages exist in the meta-analysis. For the current meta-analyses, several limitations need to be taken with cautions. Firstly, obesity is always accompanied by other complications such as coronary artery diseases and hypertension. These confounding factors needed to be adjusted in the original case–control studies. We were unable to obtain the related information. Therefore we can’t exclude a chance of the positive findings confounded by these obesity-related factors. Secondly, the significant result of *FAIM2* rs7138803 needs to be validated in the future. However, after Bonferroni’s correction by the number of testing, the association of *FAIM2* rs7138803 was unable to retain significant. Thirdly, power analysis suggested moderate power in the meta-analyses of *MTHFR* rs1801133 (power = 78.2%) and *SERPINE1* rs1799768 (power = 69.4%) The negative results of them might be caused by a lack of power in our meta-analyses. Future studies with larger samples may help clarify the contribution of these biomarkers to the risk of overweight/obesity.

Our results identified significant associations between 2 polymorphisms (*SH2B1* rs7498665 and *FAIM2* rs7138803) and overweight/obesity. Moreover, overweight/obesity is a complicated disease influenced by both genetic and environmental factors. The potential mechanism of interaction between gene and environment could be taken into consideration in the future study. Well-designed studies with large samples could help elucidate the contribution of above polymorphisms to overweight/obesity.

## Competing interests

The authors declare that they have no competing interests.

## Authors’ contribution

QH, LX and SB conceived the study idea and designed the study. FC, QL and QH reviewed the literature and performed statistical analyses. LT and HY extracted data and drafted the manuscript. SD, YM DW and MY reviewed and edited the manuscript. All authors read and approved the final manuscript.

## Authors’ information

Linlin Tang and Huadan Ye: co-first authors of this work.

## Supplementary Material

Additional file 1: Table S1 Flow diagram of selecting studies for meta-analysis. Click here for file

Additional file 2: Figure S1 Forest plots of the association studies of *UCP1* rs1800592 in our subgroup meta-analysis. Click here for file

Additional file 3: Figure S2 Forest plots of the association studies of *APOE* ϵ2/ϵ3/ϵ4. Click here for file

Additional file 4: Figure S3 Funnel plots of the studies related to *UCP1* rs1800592 by subgroup meta-analysis and *APOE* ϵ2/ϵ3/ϵ4. Click here for file

## References

[B1] Ogunbode AM Ladipo M Ajayi IO Fatiregun AA Obesity: an emerging disease Niger J Clin Pract 2011 14 4 390 394 10.4103/1119-3077.91741 22248935

[B2] Haslam DW James WP Obesity Lancet 2005 366 9492 1197 1209 10.1016/S0140-6736(05)67483-1 16198769

[B3] The situation and trends of obesity and overweight http://www.who.int/gho/ncd/risk_factors/overweight/en/index.html

[B4] Keaver L Webber L Dee A Shiely F Marsh T Balanda K Perry I Application of the UK foresight obesity model in ireland: the health and economic consequences of projected obesity trends in ireland PLoS One 2013 8 11 e79827 10.1371/journal.pone.0079827 24236162 PMC3827424

[B5] Schwenk RW Vogel H Schurmann A Genetic and epigenetic control of metabolic health Mol Metab 2013 2 4 337 347 10.1016/j.molmet.2013.09.002 24327950 PMC3854991

[B6] Latham KE Sapienza C Engel N The epigenetic lorax: gene-environment interactions in human health Epigenomics 2012 4 4 383 402 10.2217/epi.12.31 22920179 PMC3471221

[B7] Wei D Zhang X Zou H Wang L Fu B Wu X Luo Z Li X Ge J Li Y Zhu H Wang K Wang T Yang P Hou Z Wang W WW domain containing oxidoreductase induces apoptosis in gallbladder-derived malignant cell by upregulating expression of P73 and PUMA Tumour Biol 2013 35 2 1539 1550 24127039 10.1007/s13277-013-1213-1

[B8] Fukuoka H Mukai S Taniguchi T [Nutritional environment in utero and development of obesity] Nihon Rinsho 2013 71 2 237 243 23631199

[B9] Jiang D Zheng D Wang L Huang Y Liu H Xu L Liao Q Liu P Shi X Wang Z Sun L Zhou Q Li N Le Y Ye M Shao G Duan S Elevated PLA2G7 gene promoter methylation as a gender-specific marker of aging increases the risk of coronary heart disease in females PLoS One 2013 8 3 e59752 10.1371/journal.pone.0059752 23555769 PMC3610900

[B10] Tisherman SA Salvage techniques in traumatic cardiac arrest: thoracotomy, extracorporeal life support, and therapeutic hypothermia Curr Opin Crit Care 2013 19 6 594 598 24240825 10.1097/MCC.0000000000000034

[B11] Monda KL Chen GK Taylor KC Palmer C Edwards TL Lange LA Ng MC Adeyemo AA Allison MA Bielak LF Chen G Graff M Irvin MR Rhie SK Li G Liu Y Lu Y Nalls MA Sun YV Wojczynski MK Yanek LR Aldrich MC Ademola A Amos CI Bandera EV Bock CH Britton A Broeckel U Cai Q Caporaso NE A meta-analysis identifies new loci associated with body mass index in individuals of African ancestry Nat Genet 2013 45 6 690 696 10.1038/ng.2608 23583978 PMC3694490

[B12] Klenke S Kussmann M Siffert W The GNB3 C825T polymorphism as a pharmacogenetic marker in the treatment of hypertension, obesity, and depression Pharmacogenet Genomics 2011 21 9 594 606 10.1097/FPC.0b013e3283491153 21709600

[B13] Piedade MC Galhardo MS Battlehner CN Ferreira MA Caldini EG de Toledo OM Effect of ultrasound therapy on the repair of gastrocnemius muscle injury in rats Ultrasonics 2008 48 5 403 411 10.1016/j.ultras.2008.01.009 18384832

[B14] Chmurzynska A Malinowska AM Twardowska-Rajewska J Gawecki J Elderly women: homocysteine reduction by short-term folic acid supplementation resulting in increased glucose concentrations and affecting lipid metabolism (C677T MTHFR polymorphism) Nutrition 2013 29 6 841 844 10.1016/j.nut.2012.09.015 23298970

[B15] Engeli S Bohnke J Feldpausch M Gorzelniak K Janke J Batkai S Pacher P Harvey-White J Luft FC Sharma AM Jordan J Activation of the peripheral endocannabinoid system in human obesity Diabetes 2005 54 10 2838 2843 10.2337/diabetes.54.10.2838 16186383 PMC2228268

[B16] Di Marzo V Matias I Endocannabinoid control of food intake and energy balance Nat Neurosci 2005 8 5 585 589 10.1038/nn1457 15856067

[B17] Feng Q Jiang L Berg RL Antonik M MacKinney E Gunnell-Santoro J McCarty CA Wilke RA A common CNR1 (cannabinoid receptor 1) haplotype attenuates the decrease in HDL cholesterol that typically accompanies weight gain PLoS One 2010 5 12 e15779 10.1371/journal.pone.0015779 21209828 PMC3013130

[B18] Kernie SG Liebl DJ Parada LF BDNF regulates eating behavior and locomotor activity in mice EMBO J 2000 19 6 1290 1300 10.1093/emboj/19.6.1290 10716929 PMC305670

[B19] Xu B Goulding EH Zang K Cepoi D Cone RD Jones KR Tecott LH Reichardt LF Brain-derived neurotrophic factor regulates energy balance downstream of melanocortin-4 receptor Nat Neurosci 2003 6 7 736 742 10.1038/nn1073 12796784 PMC2710100

[B20] Tsuchida A Nonomura T Nakagawa T Itakura Y Ono-Kishino M Yamanaka M Sugaru E Taiji M Noguchi H Brain-derived neurotrophic factor ameliorates lipid metabolism in diabetic mice Diabetes Obes Metab 2002 4 4 262 269 10.1046/j.1463-1326.2002.00206.x 12099975

[B21] Cravatt BF Giang DK Mayfield SP Boger DL Lerner RA Gilula NB Molecular characterization of an enzyme that degrades neuromodulatory fatty-acid amides Nature 1996 384 6604 83 87 10.1038/384083a0 8900284

[B22] Lieb W Manning AK Florez JC Dupuis J Cupples LA McAteer JB Vasan RS Hoffmann U O’Donnell CJ Meigs JB Fox CS Variants in the CNR1 and the FAAH genes and adiposity traits in the community Obesity (Silver Spring) 2009 17 4 755 760 10.1038/oby.2008.608 19165169 PMC3039277

[B23] Louis SN Jackman GP Nero TL Iakovidis D Louis WJ Role of beta-adrenergic receptor subtypes in lipolysis Cardiovasc Drugs Ther 2000 14 6 565 577 10.1023/A:1007838125152 11300357

[B24] Ueta CB Fernandes GW Capelo LP Fonseca TL Maculan FD Gouveia CH Brum PC Christoffolete MA Aoki MS Lancellotti CL Kim B Bianco AC Ribeiro MO beta(1) Adrenergic receptor is key to cold- and diet-induced thermogenesis in mice J Endocrinol 2012 214 3 359 365 10.1530/JOE-12-0155 22728333 PMC4977996

[B25] Penas-Steinhardt A Tellechea ML Gomez-Rosso L Brites F Frechtel GD Poskus E Association of common variants in JAK2 gene with reduced risk of metabolic syndrome and related disorders BMC Med Genet 2011 12 166 10.1186/1471-2350-12-166 22185674 PMC3259043

[B26] Duan C Li M Rui L SH2-B promotes insulin receptor substrate 1 (IRS1)- and IRS2-mediated activation of the phosphatidylinositol 3-kinase pathway in response to leptin J Biol Chem 2004 279 42 43684 43691 10.1074/jbc.M408495200 15316008 PMC3874232

[B27] Helwig M Khorooshi RM Tups A Barrett P Archer ZA Exner C Rozman J Braulke LJ Mercer JG Klingenspor M PC1/3 and PC2 gene expression and post-translational endoproteolytic pro-opiomelanocortin processing is regulated by photoperiod in the seasonal Siberian hamster (Phodopus sungorus) J Neuroendocrinol 2006 18 6 413 425 10.1111/j.1365-2826.2006.01431.x 16684131

[B28] Farooqi IS Volders K Stanhope R Heuschkel R White A Lank E Keogh J O’Rahilly S Creemers JW Hyperphagia and early-onset obesity due to a novel homozygous missense mutation in prohormone convertase 1/3 J Clin Endocrinol Metab 2007 92 9 3369 3373 10.1210/jc.2007-0687 17595246

[B29] Dionne IJ Garant MJ Nolan AA Pollin TI Lewis DG Shuldiner AR Poehlman ET Association between obesity and a polymorphism in the beta(1)-adrenoceptor gene (Gly389Arg ADRB1) in Caucasian women Int J Obes Relat Metab Disord 2002 26 5 633 639 10.1038/sj.ijo.0801971 12032746

[B30] Naveilhan P Hassani H Canals JM Ekstrand AJ Larefalk A Chhajlani V Arenas E Gedda K Svensson L Thoren P Ernfors P Normal feeding behavior, body weight and leptin response require the neuropeptide Y Y2 receptor Nat Med 1999 5 10 1188 1193 10.1038/13514 10502824

[B31] Siddiq A Gueorguiev M Samson C Hercberg S Heude B Levy-Marchal C Jouret B Weill J Meyre D Walley A Froguel P Single nucleotide polymorphisms in the neuropeptide Y2 receptor (NPY2R) gene and association with severe obesity in French white subjects Diabetologia 2007 50 3 574 584 10.1007/s00125-006-0555-2 17235527

[B32] Somia NV Schmitt MJ Vetter DE Van Antwerp D Heinemann SF Verma IM LFG: an anti-apoptotic gene that provides protection from Fas-mediated cell death Proc Natl Acad Sci U S A 1999 96 22 12667 12672 10.1073/pnas.96.22.12667 10535980 PMC23041

[B33] Leon-Mimila P Villamil-Ramirez H Villalobos-Comparan M Villarreal-Molina T Romero-Hidalgo S Lopez-Contreras B Gutierrez-Vidal R Vega-Badillo J Jacobo-Albavera L Posadas-Romeros C Canizalez-Roman A Rio-Navarro BD Campos-Perez F Acuna-Alonzo V Aguilar-Salinas C Canizales-Quinteros S Contribution of common genetic variants to obesity and obesity-related traits in mexican children and adults PLoS One 2013 8 8 e70640 10.1371/journal.pone.0070640 23950976 PMC3738539

[B34] Eriksson P Reynisdottir S Lonnqvist F Stemme V Hamsten A Arner P Adipose tissue secretion of plasminogen activator inhibitor-1 in non-obese and obese individuals Diabetologia 1998 41 1 65 71 10.1007/s001250050868 9498632

[B35] Ferretti G Bacchetti T Masciangelo S Bicchiega V HDL-paraoxonase and membrane lipid peroxidation: a comparison between healthy and obese subjects Obesity (Silver Spring) 2010 18 6 1079 1084 10.1038/oby.2009.338 19834469

[B36] Pachocka LM Wlodarczyk M Nowicka G Klosiewicz-Latoszek L Wolanska D Stolarska I [CETP gene TaqIB polymorphism and plasma lipids in patients with overweight and obesity] Rocz Panstw Zakl Hig 2012 63 2 149 154 22928361

[B37] Chapman MJ Le Goff W Guerin M Kontush A Cholesteryl ester transfer protein: at the heart of the action of lipid-modulating therapy with statins, fibrates, niacin, and cholesteryl ester transfer protein inhibitors Eur Heart J 2010 31 2 149 164 10.1093/eurheartj/ehp399 19825813 PMC2806550

[B38] Cannon B Nedergaard J Brown adipose tissue: function and physiological significance Physiol Rev 2004 84 1 277 359 10.1152/physrev.00015.2003 14715917

[B39] Miyaki K Sutani S Kikuchi H Takei I Murata M Watanabe K Omae K Increased risk of obesity resulting from the interaction between high energy intake and the Trp64Arg polymorphism of the beta3-adrenergic receptor gene in healthy Japanese men J Epidemiol 2005 15 6 203 210 10.2188/jea.15.203 16276029 PMC7904380

[B40] Dalgaard LT Pedersen O Uncoupling proteins: functional characteristics and role in the pathogenesis of obesity and Type II diabetes Diabetologia 2001 44 8 946 965 10.1007/s001250100596 11484071

[B41] Costford S Gowing A Harper ME Mitochondrial uncoupling as a target in the treatment of obesity Curr Opin Clin Nutr Metab Care 2007 10 6 671 678 10.1097/MCO.0b013e3282f0dbe4 18089946

[B42] Oram JF Heinecke JW ATP-binding cassette transporter A1: a cell cholesterol exporter that protects against cardiovascular disease Physiol Rev 2005 85 4 1343 1372 10.1152/physrev.00005.2005 16183915

[B43] Ghorbanian B Ravassi A Kordi MR Hedayati M The Effects of Rope Training on Lymphocyte ABCA1 Expression, Plasma ApoA-I and HDL-c in Boy Adolescents Int J Endocrinol Metab 2013 11 2 76 81 23825977 10.5812/ijem.8178 PMC3693670

[B44] Mahley RW Apolipoprotein E: cholesterol transport protein with expanding role in cell biology Science 1988 240 4852 622 630 10.1126/science.3283935 3283935

[B45] Wang J Perrard XD Perrard JL Mukherjee A Rosales C Chen Y Smith CW Pownall HJ Ballantyne CM Wu H ApoE and the role of very low density lipoproteins in adipose tissue inflammation Atherosclerosis 2012 223 2 342 349 10.1016/j.atherosclerosis.2012.06.003 22770993 PMC3411924

[B46] Xu Z Yu L Zhang X Association between the hOGG1 Ser326Cys polymorphism and lung cancer susceptibility: a meta-analysis based on 22,475 subjects Diagn Pathol 2013 8 144 10.1186/1746-1596-8-144 23971971 PMC3853705

[B47] de Matos LL Del Giglio AB Matsubayashi CO de Lima Farah M Del Giglio A da Silva Pinhal MA Expression of CK-19, galectin-3 and HBME-1 in the differentiation of thyroid lesions: systematic review and diagnostic meta-analysis Diagn Pathol 2012 7 97 10.1186/1746-1596-7-97 22888980 PMC3523001

[B48] Meta-analysis http://en.wikipedia.org/wiki/Meta-analysis

[B49] The criteria for overweight or obesity in children and adolescents http://www.who.int/dietphysicalactivity/childhood_what/en/index.html

[B50] The criteria for overweight or obesity in children http://www.who.int/growthref/who2007_bmi_for_age/en/index.html

[B51] Jiang H Sun MW Hefright B Chen W Lu CD Zeng J Efficacy of hypocaloric parenteral nutrition for surgical patients: a systematic review and meta-analysis Clin Nutr 2011 30 6 730 737 10.1016/j.clnu.2011.05.006 21704437

[B52] Dupont WD Plummer WD Jr Power and sample size calculations. A review and computer program Control Clin Trials 1990 11 2 116 128 10.1016/0197-2456(90)90005-M 2161310

[B53] Srivastava N Achyut BR Prakash J Agarwal CG Pant DC Mittal B Association of cholesteryl ester transfer protein (TaqIB) and apolipoprotein E (HhaI) gene variants with obesity Mol Cell Biochem 2008 314 1–2 171 177 18454345 10.1007/s11010-008-9778-5

[B54] Maures TJ Kurzer JH Carter-Su C SH2B1 (SH2-B) and JAK2: a multifunctional adaptor protein and kinase made for each other Trends Endocrinol Metab 2007 18 1 38 45 10.1016/j.tem.2006.11.007 17140804

[B55] Speakman JR Functional analysis of seven genes linked to body mass index and adiposity by genome-wide association studies: a review Hum Hered 2013 75 2–4 57 79 24081222 10.1159/000353585

[B56] Richard AJ Stephens JM Emerging roles of JAK-STAT signaling pathways in adipocytes Trends Endocrinol Metab 2011 22 8 325 332 10.1016/j.tem.2011.03.007 21561789 PMC3149764

[B57] Ren D Li M Rui L Identification of SH2-B as a key regulator of leptin sensitivity, energy balance, and body weight in mice Cell Metab 2005 2 2 95 104 10.1016/j.cmet.2005.07.004 16098827

[B58] Paternoster L Evans DM Nohr EA Holst C Gaborieau V Brennan P Gjesing AP Grarup N Witte DR Jorgensen T Linneberg A Lauritzen T Sandbaek A Hansen T Pedersen O Elliott KS Kemp JP St Pourcain B McMahon G Zelenika D Hager J Lathrop M Timpson NJ Smith GD Sorensen TI Genome-wide population-based association study of extremely overweight young adults–the GOYA study PLoS One 2011 6 9 e24303 10.1371/journal.pone.0024303 21935397 PMC3174168

[B59] Hotta K Nakamura M Nakamura T Matsuo T Nakata Y Kamohara S Miyatake N Kotani K Komatsu R Itoh N Mineo I Wada J Masuzaki H Yoneda M Nakajima A Funahashi T Miyazaki S Tokunaga K Kawamoto M Ueno T Hamaguchi K Tanaka K Yamada K Hanafusa T Oikawa S Yoshimatsu H Nakao K Sakata T Matsuzawa Y Kamatani N Association between obesity and polymorphisms in SEC16B, TMEM18, GNPDA2, BDNF, FAIM2 and MC4R in a Japanese population J Hum Genet 2009 54 12 727 731 10.1038/jhg.2009.106 19851340

[B60] Li C Qiu X Yang N Gao J Rong Y Xiong C Zheng F Common rs7138803 variant of FAIM2 and obesity in Han Chinese BMC Cardiovasc Disord 2013 13 56 10.1186/1471-2261-13-56 23924573 PMC3765134

[B61] Liu Y Tang W Wang J Xie L Li T He Y Qin X Li S Clinicopathological and prognostic significance of S100A4 overexpression in colorectal cancer: a meta-analysis Diagn Pathol 2013 8 1 181 10.1186/1746-1596-8-181 24188373 PMC3833630

[B62] Wang Z Zhang Y Kong X Li S Hu Y Wang R Li Y Lu C Lin N Chen W Association of a polymorphism in PON-1 gene with steroid-induced osteonecrosis of femoral head in Chinese Han population Diagn Pathol 2013 8 1 186 10.1186/1746-1596-8-186 24206655 PMC4226244

[B63] Zhang Y Wang R Li S Kong X Wang Z Chen W Lin N Genetic polymorphisms in plasminogen activator inhibitor-1 predict susceptibility to steroid-induced osteonecrosis of the femoral head in Chinese population Diagn Pathol 2013 8 1 169 10.1186/1746-1596-8-169 24135164 PMC4016530

