# Supplementary material for: Meta-analyses between 18 candidate genetic markers and overweight/obesity
Source: Diagn Pathol. 2014 Mar 12;9:56. doi: 10.1186/1746-1596-9-56 (PMC4008255; doi:10.1186/1746-1596-9-56)
Supplement: Additional file 1: Table S1 — Flow diagram of selecting studies for meta-analysis. [file 1746-1596-9-56-S1.docx]

Supplemental table1: Flow diagram of selecting studies for meta-analysis

Additional Chinese records identified through other sources of CNKI and Wanfang from 1990 to 2013 (n = 89)

Records after duplicates removed

(n = 7,750)

Records identified through database searching of PubMed, Embase, and Web of Science from1990 to 2013 (n = 7,661)

Exclusion of 2,256 articles about genes which had been done meta-analysis

Records screened
(n = 5,494)

Exclusion of 5,421 articles as follows:

1. without at least three case-control genetic polymorphism studies
2. without investigating the polymorphism of gene

Articles included in qualitative synthesis (n = 71)

Meta-analyses of overweight/obesity: 8 publications of *GNB3* (rs5443) ^[^[^1-8^](#_ENREF_1)^]^, 3 publications of *MTHFR* (rs1801133)^[^[^9-11^](#_ENREF_9)^]^, 3 publications of *CNR1* (rs806381)^[^[^12-14^](#_ENREF_12)^]^, 6 publications of *BDNF* (rs6265)^[^[^15-20^](#_ENREF_15)^]^, 6 publications of *FAAH* (rs324420)^[^[^21-26^](#_ENREF_21)^]^, 4 publications of *ADRB1* (rs1801253)^[^[^27-30^](#_ENREF_27)^]^, 5 publications of *SH2B1* (rs7498665)^[^[^16^](#_ENREF_16)^,^ [^18^](#_ENREF_18)^,^ [^31-33^](#_ENREF_31)^]^, 5 publications of *PCSK1*(rs6232)^[^[^16^](#_ENREF_16)^,^ [^34-37^](#_ENREF_34)^]^, 5 publications of *PCSK1* (rs6235)^[^[^34-38^](#_ENREF_34)^]^, 4 publications of *NPY2R3* (rs1047214)^[^[^39-42^](#_ENREF_39)^]^, 5 publications of *FAIM2* (rs7138803)^[^[^16-18^](#_ENREF_16)^,^ [^43^](#_ENREF_43)^,^ [^44^](#_ENREF_44)^]^, 7 publications of *SERPINE1* (rs1799768)^[^[^45-51^](#_ENREF_45)^]^, 3 publications of *PON1* (rs854560)^[^[^52-54^](#_ENREF_52)^]^, 3 publications of *PON1* (rs662)^[^[^52-54^](#_ENREF_52)^]^， 4 publications of *CETP* (TaqIB)^[^[^55-58^](#_ENREF_55)^]^, 8 publications of *UCP1* (rs1800592)^[^[^59-66^](#_ENREF_59)^]^, 3 publications of *ABCA1* (rs2230806) ^[^[^55^](#_ENREF_55)^,^ [^67^](#_ENREF_67)^,^ [^68^](#_ENREF_68)^]^, 5 publications of *APOE* (ε2/ε3/ε4)^[^[^57^](#_ENREF_57)^,^ [^69-72^](#_ENREF_69)^]^.

Reference

1. Hsiao, T.J., Hwang Y., Liu C.H., Chang H.M. and Lin E., Association of the C825T polymorphism in the GNB3 gene with obesity and metabolic phenotypes in a Taiwanese population*,* *Genes Nutr* **8**, 2013, 137-44.

2. Wang, X., Bai H., Fan P., Liu R., Liu Y. and Liu B., [Analysis of the GNB3 gene 825C/T polymorphism in non-obese and obese Chinese]*,* *Zhonghua Yi Xue Yi Chuan Xue Za Zhi* **25**, 2008, 670-4.

3. Suwazono, Y., Okubo Y., Kobayashi E., Miura K., Morikawa Y., Ishizaki M., et al., Lack of association between human G-protein beta3 subunit variant and overweight in Japanese workers*,* *Obes Res* **12**, 2004, 4-8.

4. Hinney, A., Geller F., Neupert T., Sommerlad C., Gerber G., Gorg T., et al., No evidence for involvement of alleles of the 825-C/T polymorphism of the G-protein subunit beta 3 in body weight regulation*,* *Exp Clin Endocrinol Diabetes* **109**, 2001, 402-5.

5. Ohshiro, Y., Ueda K., Wakasaki H., Takasu N. and Nanjo K., Analysis of 825C/T polymorphism of G proteinbeta3 subunit in obese/diabetic Japanese*,* *Biochem Biophys Res Commun* **286**, 2001, 678-80.

6. Benjafield, A.V., Lin R.C., Dalziel B., Gosby A.K., Caterson I.D. and Morris B.J., G-protein beta3 subunit gene splice variant in obesity and overweight*,* *Int J Obes Relat Metab Disord* **25**, 2001, 777-80.

7. Siffert, W., Rosskopf D. and Erbel R., [Genetic polymorphism of the G-protein beta3 subunit, obesity and essential hypertension]*,* *Herz* **25**, 2000, 26-33.

8. Siffert, W., Forster P., Jockel K.H., Mvere D.A., Brinkmann B., Naber C., et al., Worldwide ethnic distribution of the G protein beta3 subunit 825T allele and its association with obesity in Caucasian, Chinese, and Black African individuals*,* *J Am Soc Nephrol* **10**, 1999, 1921-30.

9. Yin, R.X., Wu D.F., Miao L., Aung L.H., Cao X.L., Yan T.T., et al., Several genetic polymorphisms interact with overweight/obesity to influence serum lipid levels*,* *Cardiovasc Diabetol* **11**, 2012, 123.

10. Tavakkoly Bazzaz, J., Shojapoor M., Nazem H., Amiri P., Fakhrzadeh H., Heshmat R., et al., Methylenetetrahydrofolate reductase gene polymorphism in diabetes and obesity*,* *Mol Biol Rep* **37**, 2010, 105-9.

11. Terruzzi, I., Senesi P., Fermo I., Lattuada G. and Luzi L., Are genetic variants of the methyl group metabolism enzymes risk factors predisposing to obesity?*,* *J Endocrinol Invest* **30**, 2007, 747-53.

12. Zhuang, M., Yang Y., Cao F., Lu M., Wang X., Zhang J., et al., Associations of variants of CNR1 with obesity and obesity-related traits in Chinese women*,* *Gene* **495**, 2012, 194-8.

13. Jaeger, J.P., Mattevi V.S., Callegari-Jacques S.M. and Hutz M.H., Cannabinoid type-1 receptor gene polymorphisms are associated with central obesity in a Southern Brazilian population*,* *Dis Markers* **25**, 2008, 67-74.

14. Benzinou, M., Chevre J.C., Ward K.J., Lecoeur C., Dina C., Lobbens S., et al., Endocannabinoid receptor 1 gene variations increase risk for obesity and modulate body mass index in European populations*,* *Hum Mol Genet* **17**, 2008, 1916-21.

15. Skledar, M., Nikolac M., Dodig-Curkovic K., Curkovic M., Borovecki F. and Pivac N., Association between brain-derived neurotrophic factor Val66Met and obesity in children and adolescents*,* *Prog Neuropsychopharmacol Biol Psychiatry* **36**, 2012, 136-40.

16. Rouskas, K., Kouvatsi A., Paletas K., Papazoglou D., Tsapas A., Lobbens S., et al., Common variants in FTO, MC4R, TMEM18, PRL, AIF1, and PCSK1 show evidence of association with adult obesity in the Greek population*,* *Obesity (Silver Spring)* **20**, 2012, 389-95.

17. Xi, B., Wang C., Wu L., Zhang M., Shen Y., Zhao X., et al., Influence of physical inactivity on associations between single nucleotide polymorphisms and genetic predisposition to childhood obesity*,* *Am J Epidemiol* **173**, 2011, 1256-62.

18. Hotta, K., Nakamura M., Nakamura T., Matsuo T., Nakata Y., Kamohara S., et al., Association between obesity and polymorphisms in SEC16B, TMEM18, GNPDA2, BDNF, FAIM2 and MC4R in a Japanese population*,* *J Hum Genet* **54**, 2009, 727-31.

19. Marti, A., Santos J.L., Gratacos M., Moreno-Aliaga M.J., Maiz A., Martinez J.A., et al., Association between leptin receptor (LEPR) and brain-derived neurotrophic factor (BDNF) gene variants and obesity: a case-control study*,* *Nutr Neurosci* **12**, 2009, 183-8.

20. Friedel, S., Horro F.F., Wermter A.K., Geller F., Dempfle A., Reichwald K., et al., Mutation screen of the brain derived neurotrophic factor gene (BDNF): identification of several genetic variants and association studies in patients with obesity, eating disorders, and attention-deficit/hyperactivity disorder*,* *Am J Med Genet B Neuropsychiatr Genet* **132B**, 2005, 96-9.

21. Muller, T.D., Bronner G., Wandolski M., Carrie J., Nguyen T.T., Greene B.H., et al., Mutation screen and association studies for the fatty acid amide hydrolase (FAAH) gene and early onset and adult obesity*,* *BMC Med Genet* **11**, 2010, 2.

22. Papazoglou, D., Panagopoulos I., Papanas N., Gioka T., Papadopoulos T., Papathanasiou P., et al., The fatty acid amide hydrolase (FAAH) Pro129Thr polymorphism is not associated with severe obesity in Greek subjects*,* *Horm Metab Res* **40**, 2008, 907-10.

23. Monteleone, P., Tortorella A., Martiadis V., Di Filippo C., Canestrelli B. and Maj M., The cDNA 385C to A missense polymorphism of the endocannabinoid degrading enzyme fatty acid amide hydrolase (FAAH) is associated with overweight/obesity but not with binge eating disorder in overweight/obese women*,* *Psychoneuroendocrinology* **33**, 2008, 546-50.

24. Durand, E., Lecoeur C., Delplanque J., Benzinou M., Degraeve F., Boutin P., et al., Evaluating the association of FAAH common gene variation with childhood, adult severe obesity and type 2 diabetes in the French population*,* *Obes Facts* **1**, 2008, 305-9.

25. Jensen, D.P., Andreasen C.H., Andersen M.K., Hansen L., Eiberg H., Borch-Johnsen K., et al., The functional Pro129Thr variant of the FAAH gene is not associated with various fat accumulation phenotypes in a population-based cohort of 5,801 whites*,* *J Mol Med (Berl)* **85**, 2007, 445-9.

26. Sipe, J.C., Waalen J., Gerber A. and Beutler E., Overweight and obesity associated with a missense polymorphism in fatty acid amide hydrolase (FAAH)*,* *Int J Obes (Lond)* **29**, 2005, 755-9.

27. Ohshiro, Y., Hayashi M., Yabiku K., Ueda K., Wakasaki H., Ishigame M., et al., Mutations in the beta1 adrenergic receptor gene and massive obesity in Japanese*,* *Diabetes Res Clin Pract* **80**, 2008, 213-7.

28. Gjesing, A.P., Andersen G., Albrechtsen A., Glumer C., Borch-Johnsen K., Jorgensen T., et al., Studies of associations between the Arg389Gly polymorphism of the beta1-adrenergic receptor gene (ADRB1) and hypertension and obesity in 7677 Danish white subjects*,* *Diabet Med* **24**, 2007, 392-7.

29. Tafel, J., Branscheid I., Skwarna B., Schlimme M., Morcos M., Algenstaedt P., et al., Variants in the human beta 1-, beta 2-, and beta 3-adrenergic receptor genes are not associated with morbid obesity in children and adolescents*,* *Diabetes Obes Metab* **6**, 2004, 452-5.

30. Ryden, M., Hoffstedt J., Eriksson P., Bringman S. and Arner P., The Arg 389 Gly beta1-adrenergic receptor gene polymorphism and human fat cell lipolysis*,* *Int J Obes Relat Metab Disord* **25**, 2001, 1599-603.

31. Volckmar, A.L., Bolze F., Jarick I., Knoll N., Scherag A., Reinehr T., et al., Mutation screen in the GWAS derived obesity gene SH2B1 including functional analyses of detected variants*,* *BMC Med Genomics* **5**, 2012, 65.

32. Beckers, S., Zegers D., Van Gaal L.F. and Van Hul W., Replication of the SH2B1 rs7498665 association with obesity in a Belgian study population*,* *Obes Facts* **4**, 2011, 473-7.

33. Shi, J., Long J., Gao Y.T., Lu W., Cai Q., Wen W., et al., Evaluation of genetic susceptibility loci for obesity in Chinese women*,* *Am J Epidemiol* **172**, 2010, 244-54.

34. Dusatkova, L., Zamrazilova H., Sedlackova B., Vcelak J., Hlavaty P., Aldhoon Hainerova I., et al., Association of obesity susceptibility gene variants with metabolic syndrome and related traits in 1,443 czech adolescents*,* *Folia Biol (Praha)* **59**, 2013, 123-33.

35. Choquet, H., Kasberger J., Hamidovic A. and Jorgenson E., Contribution of common PCSK1 genetic variants to obesity in 8,359 subjects from multi-ethnic American population*,* *PLoS One* **8**, 2013, e57857.

36. Villalobos-Comparan, M., Villamil-Ramirez H., Villarreal-Molina T., Larrieta-Carrasco E., Leon-Mimila P., Romero-Hidalgo S., et al., PCSK1 rs6232 is associated with childhood and adult class III obesity in the Mexican population*,* *PLoS One* **7**, 2012, e39037.

37. Happe, F. and Vital P., What aspects of autism predispose to talent?*,* *Philos Trans R Soc Lond B Biol Sci* **364**, 2009, 1369-75.

38. Hsiao, T.J., Hwang Y., Chang H.M. and Lin E., Association of the rs6235 variant in the proprotein convertase subtilisin/kexin type 1 (PCSK1) gene with obesity and related traits in a Taiwanese population*,* *Gene* **533**, 2014, 32-7.

39. Zhang, J., Wang H.J. and Ma J., [Association between obesity and the polymorphism of neuropeptide Y2 receptor gene in children and adolescents]*,* *Zhonghua Liu Xing Bing Xue Za Zhi* **30**, 2009, 695-8.

40. Wang, H.J., Wermter A.K., Nguyen T.T., Scherag A., Reichwald K., Waldenmaier B., et al., No association of sequence variants in the neuropeptide Y2 receptor (NPY2R) gene with early onset obesity in Germans*,* *Horm Metab Res* **39**, 2007, 840-4.

41. Siddiq, A., Gueorguiev M., Samson C., Hercberg S., Heude B., Levy-Marchal C., et al., Single nucleotide polymorphisms in the neuropeptide Y2 receptor (NPY2R) gene and association with severe obesity in French white subjects*,* *Diabetologia* **50**, 2007, 574-84.

42. Torekov, S.S., Larsen L.H., Andersen G., Albrechtsen A., Glumer C., Borch-Johnsen K., et al., Variants in the 5' region of the neuropeptide Y receptor Y2 gene (NPY2R) are associated with obesity in 5,971 white subjects*,* *Diabetologia* **49**, 2006, 2653-8.

43. Zhao, X.Y., Zhang M.X., Cheng H., Yan Y.K., Wu L.J., Shen Y., et al., [Risk of obesity-related gene polymorphism on the incidence and durative of childhood obesity]*,* *Zhonghua Liu Xing Bing Xue Za Zhi* **34**, 2013, 560-5.

44. Li, C., Qiu X., Yang N., Gao J., Rong Y., Xiong C., et al., Common rs7138803 variant of FAIM2 and obesity in Han Chinese*,* *BMC Cardiovasc Disord* **13**, 2013, 56.

45. Wingeyer, S.D., Graffigna M.N., Belli S.H., Benetucci J. and de Larranaga G.F., Role of -675 4G/5G in the plasminogen activator inhibitor-1 gene and -308G/A tumor necrosis factor-alpha gene polymorphisms in obese Argentinean patients*,* *Genet Test Mol Biomarkers* **16**, 2012, 372-5.

46. Espino, A., Villagran A., Vollrath V., Hanckes P., Salas R., Farah A., et al., Plasminogen activator inhibitor type 1 serum levels and 4G/5G gene polymorphism in morbidly obese Hispanic patients with non-alcoholic fatty liver disease*,* *Ann Hepatol* **10**, 2011, 493-501.

47. Kinik, S.T., Ozbek N., Yuce M., Yazici A.C., Verdi H. and Atac F.B., PAI-1 gene 4G/5G polymorphism, cytokine levels and their relations with metabolic parameters in obese children*,* *Thromb Haemost* **99**, 2008, 352-6.

48. Sola, E., Vaya A., Espana F., Castello R., Ramon L.A., Hernandez-Mijares A., et al., Plasminogen activator inhibitor-1 levels in severe and morbid obesity. Effect of weight loss and influence of 4G/5G polymorphism*,* *Thromb Res* **122**, 2008, 320-7.

49. Berberoglu, M., Evliyaoglu O., Adiyaman P., Ocal G., Ulukol B., Simsek F., et al., Plasminogen activator inhibitor-1 (PAI-1) gene polymorphism (-675 4G/5G) associated with obesity and vascular risk in children*,* *J Pediatr Endocrinol Metab* **19**, 2006, 741-8.

50. Hoffstedt, J., Andersson I.L., Persson L., Isaksson B. and Arner P., The common -675 4G/5G polymorphism in the plasminogen activator inhibitor -1 gene is strongly associated with obesity*,* *Diabetologia* **45**, 2002, 584-7.

51. Sartori, M.T., Vettor R., De Pergola G., De Mitrio V., Saggiorato G., Della Mea P., et al., Role of the 4G/5G polymorphism of PaI-1 gene promoter on PaI-1 levels in obese patients: influence of fat distribution and insulin-resistance*,* *Thromb Haemost* **86**, 2001, 1161-9.

52. Ruperez, A.I., Lopez-Guarnido O., Gil F., Olza J., Gil-Campos M., Leis R., et al., Paraoxonase 1 activities and genetic variation in childhood obesity*,* *Br J Nutr* **110**, 2013, 1639-47.

53. Martinez-Salazar, M.F., Almenares-Lopez D., Garcia-Jimenez S., Sanchez-Aleman M.A., Juantorena-Ugas A., Rios C., et al., Relationship between the paraoxonase (PON1) L55M and Q192R polymorphisms and obesity in a Mexican population: a pilot study*,* *Genes Nutr* **6**, 2011, 361-8.

54. Veiga, L., Silva-Nunes J., Melao A., Oliveira A., Duarte L. and Brito M., Q192R polymorphism of the paraoxonase-1 gene as a risk factor for obesity in Portuguese women*,* *Eur J Endocrinol* **164**, 2011, 213-8.

55. Huang, Y., Wu Y., Liu R., Fan P., Zhang J., Wang F., et al., Differential effect of ATP binding cassette transporter A1 R219K and cholesteryl ester transfer protein TaqIB genotypes on HDL-C levels in overweight/obese and non-obese Chinese subjects*,* *Acta Cardiol* **66**, 2011, 231-7.

56. Ruan, X., Ma L., Wang S., Lindpaintner K., Liu X., Wang B., et al., TAQIB and I405V polymorphisms of CETP are moderately associated with obesity risk in the Chinese adult population*,* *Acta Diabetol* **47**, 2010, 217-24.

57. Srivastava, N., Achyut B.R., Prakash J., Agarwal C.G., Pant D.C. and Mittal B., Association of cholesteryl ester transfer protein (TaqIB) and apolipoprotein E (HhaI) gene variants with obesity*,* *Mol Cell Biochem* **314**, 2008, 171-7.

58. Huang, Z.Y., Guo H.W., Xu Z.H. and Xue K., [Association of gene polymorphism at cholesterol ester transfer protein locus with obesity and response to dietary intervention in obesity]*,* *Wei Sheng Yan Jiu* **35**, 2006, 447-9.

59. Shen, Z.N., Wang X.S., Bai H., Fan P., Liu R., Liu Y., et al., [Analysis of -3826A/G polymorphism in the promoter of the uncoupling protein-1 gene in Chinese non-obese and obese populations]*,* *Zhonghua Yi Xue Yi Chuan Xue Za Zhi* **26**, 2009, 555-61.

60. Mottagui-Tabar, S., Hoffstedt J., Brookes A.J., Jiao H., Arner P. and Dahlman I., Association of ADRB1 and UCP3 gene polymorphisms with insulin sensitivity but not obesity*,* *Horm Res* **69**, 2008, 31-6.

61. Ramis, J.M., Gonzalez-Sanchez J.L., Proenza A.M., Martinez-Larrad M.T., Fernandez-Perez C., Palou A., et al., The Arg64 allele of the beta 3-adrenoceptor gene but not the -3826G allele of the uncoupling protein 1 gene is associated with increased leptin levels in the Spanish population*,* *Metabolism* **53**, 2004, 1411-6.

62. Forga, L., Corbalan M., Marti A., Fuentes C., Martinez-Gonzalez M.A. and Martinez A., [Influence of the polymorphism 03826 A --> G in the UCP1 gene on the components of metabolic syndrome]*,* *An Sist Sanit Navar* **26**, 2003, 231-6.

63. Nieters, A., Becker N. and Linseisen J., Polymorphisms in candidate obesity genes and their interaction with dietary intake of n-6 polyunsaturated fatty acids affect obesity risk in a sub-sample of the EPIC-Heidelberg cohort*,* *Eur J Nutr* **41**, 2002, 210-21.

64. Kiec-Wilk, B., Wybranska I., Malczewska-Malec M., Leszczynska-Golabek L., Partyka L., Niedbal S., et al., Correlation of the -3826A >G polymorphism in the promoter of the uncoupling protein 1 gene with obesity and metabolic disorders in obese families from southern Poland*,* *J Physiol Pharmacol* **53**, 2002, 477-90.

65. Proenza, A.M., Poissonnet C.M., Ozata M., Ozen S., Guran S., Palou A., et al., Association of sets of alleles of genes encoding beta3-adrenoreceptor, uncoupling protein 1 and lipoprotein lipase with increased risk of metabolic complications in obesity*,* *Int J Obes Relat Metab Disord* **24**, 2000, 93-100.

66. Gagnon, J., Lago F., Chagnon Y.C., Perusse L., Naslund I., Lissner L., et al., DNA polymorphism in the uncoupling protein 1 (UCP1) gene has no effect on obesity related phenotypes in the Swedish Obese Subjects cohorts*,* *Int J Obes Relat Metab Disord* **22**, 1998, 500-5.

67. Kitjaroentham, A., Hananantachai H., Tungtrongchitr A., Pooudong S. and Tungtrongchitr R., R219K polymorphism of ATP binding cassette transporter A1 related with low HDL in overweight/obese Thai males*,* *Arch Med Res* **38**, 2007, 834-8.

68. Porchay, I., Pean F., Bellili N., Royer B., Cogneau J., Chesnier M.C., et al., ABCA1 single nucleotide polymorphisms on high-density lipoprotein-cholesterol and overweight: the D.E.S.I.R. study*,* *Obesity (Silver Spring)* **14**, 2006, 1874-9.

69. Zhang, J., Xuemei Z., Fan P., Liu R., Huang Y., Liang S., et al., Distribution and effect of apo E genotype on plasma lipid and apolipoprotein profiles in overweight/obese and nonobese Chinese subjects*,* *J Clin Lab Anal* **26**, 2012, 200-5.

70. Ergun, M.A., Karaoguz M.Y., Koc A., Camurdan O., Bideci A., Yazici A.C., et al., The apolipoprotein E gene and Taq1A polymorphisms in childhood obesity*,* *Genet Test Mol Biomarkers* **14**, 2010, 343-5.

71. Guerra, A., Rego C., Castro E.M., Seixas S. and Rocha J., Influence of apolipoprotein e polymorphism on cardiovascular risk factors in obese children*,* *Ann Nutr Metab* **47**, 2003, 49-54.

72. Przybylska, B., Wyrzykowski Z., Wyrzykowska K. and Karasek M., Ultrastructure of pig pinealocytes in various stages of the sexual cycle: a quantitative study*,* *Cytobios* **64**, 1990, 7-14.
